# The effects of physical exercise on cardiometabolic outcomes in women with polycystic ovary syndrome not taking the oral contraceptive pill: a systematic review and meta-analysis

**DOI:** 10.1007/s40200-019-00425-y

**Published:** 2019-07-29

**Authors:** Amie Woodward, David Broom, Deborah Harrop, Ian Lahart, Anouska Carter, Caroline Dalton, Mostafa Metwally, Markos Klonizakis

**Affiliations:** 1 Faculty of Health and Wellbeing, Sheffield Hallam University, Collegiate Crescent, Sheffield, S10 2BP UK; 2 Institute of Human Science, University of Wolverhampton, Wolverhampton, WV1 1LY UK; 3 Faculty of Health and Wellbeing, Sheffield Hallam University, Howard Street, Sheffield, S1 1WB UK; 4 Jessop Wing, Sheffield Teaching Hospitals NHS Foundation Trust, Tree Root Walk, Sheffield, S10 2SF UK

**Keywords:** Polycystic ovary syndrome, Exercise, Cardiovascular disease, Metabolism

## Abstract

**Purpose:**

Women with polycystic ovary syndrome (PCOS) exhibit many metabolic abnormalities that are associated with an increased cardiovascular disease risk. Exercise may promote improvements in lipid profile and insulin sensitivity in women with PCOS. There is however, a knowledge gap on the optimal dose of exercise, regarding duration, intensity, type, and frequency of exercise. The aim of this systematic review and meta-analysis was to define effective types of exercise to improve cardiometabolic profile in PCOS.

**Methods:**

We included randomised controlled trials (RCT), quasi-RCT, and controlled clinical trials focusing on reproductive-aged women diagnosed with PCOS. Eligible interventions included those with at least two weeks of supervised exercise sessions. Primary outcomes were blood lipids, blood glucose, blood pressure, measures of abdominal adiposity, and inflammation markers. Secondary outcomes were total and free testosterone, sex hormone binding globulin, and measures of insulin resistance. Nine electronic databases were searched from inception to present for English language publications. The Cochrane Risk Assessment tool was used to assess bias in the included studies. Outcomes were quantitatively synthesised and a meta- analysis was performed. Pooled effect estimates and 95% confidence intervals were presented.

**Results:**

This systematic review identified three trials, including 231 participants with PCOS, that examined the effect of structured, supervised exercise on cardiometabolic outcomes. Analysis of pooled data indicated statistical favourable effects of exercise on total cholesterol, fasting glucose, waist circumference and waist-to-hip ratio, systolic blood pressure, C-reactive protein, total testosterone, and sex hormone binding globulin using post-intervention scores.

**Conclusions:**

Moderate aerobic exercise interventions ≥3 months in duration, with a frequency of 3/week for at least 30-min, may have favourable effects on various cardiometabolic risk factors in women with PCOS. However, results should be interpreted with caution. Many of the outcomes were based on studies with serious methodological limitations, and only one “gold-standard” RCT was identified.

PROSPERO ID: CRD42018086117.

**Electronic supplementary material:**

The online version of this article (10.1007/s40200-019-00425-y) contains supplementary material, which is available to authorized users.

## Background

Polycystic ovary syndrome (PCOS) is a common complex hormonal and metabolic condition [1]. The now internationally accepted Rotterdam Criteria, derived by the European Society of Human Reproduction and Embryology (ESHRE) and the American Society for Reproductive Medicine (ASRM), requires that women present with at least two of the three signs/symptoms (clinical or biochemical hyperandrogenism, anovulation or oligomenorrhea, and polycystic ovaries) to receive a diagnosis, in the absence of other pathologies that can promote these symptoms [2].

The metabolic complications associated with an increased cardiovascular disease (CVD) risk in PCOS, independent of obesity [3], include insulin resistance, impaired glucose tolerance (IGT), dyslipidemia, type 2 diabetes (T2D), hypertension, subclinical atherosclerosis, and a two to four-fold higher prevalence of metabolic syndrome compared to body mass index (BMI)-matched women [4–7]. Dyslipidemia, characterised by high triglyceride (TG) and low high-density lipoprotein (HDL) concentrations, is prevalent in up to 70% of women with PCOS [5].

Inflammatory markers that are implicated in the mediation of CVD may be elevated in women with PCOS [8]. These markers range from high-sensitivity C-reactive protein [9, 10] to increased white cell count, neutrophil/lymphocyte ratio, tumour-necrosis factor-alpha (TNF-a) and interleukin-6 (IL-6) [10–13]. Moreover, a 2012 review indicates that various studies have reported that carotid intima-media thickness (cIMT), a marker of subclinical atherosclerosis, is higher in women with PCOS in comparison to controls [14].

Hyperandrogenism is associated with hyperinsulinemic states because insulin has the capacity to act as a co-gonadotrophin, thus stimulating ovarian androgen production [15]. The increased circulating androgens may then contribute to inflammation by promoting adipocyte hypertrophy and stimulating mononuclear cells to release TNF-a and IL-6 [16]. In addition, hyperandrogenism may then promote abdominal fat accumulation and further exacerbate insulin resistance. Phenotypes that present with hyperandrogenism may therefore have a worse metabolic profile despite comparable distributions of body weight [17, 18].

Lifestyle interventions and modifications are widely considered to be a cornerstone of PCOS treatment for cardiometabolic symptoms [19, 20]. Exercise interventions in PCOS have promoted improvements in lipid profile, ovulation, and insulin sensitivity by up to 30% in women with PCOS, independent of weight loss, within 12 weeks [21]. This indicates that the increased CVD risk factors associated with PCOS are not solely attributed to obesity, and lean women with PCOS can still benefit from exercise to improve their cardiometabolic profile.

There currently lacks guidance on which exercise interventions are effective for differing phenotypes, regarding duration, type of exercise and frequency of exercise sessions. Subsequently, the objective of this systematic review and meta-analysis is to define regimes of exercise interventions, which could improve the cardiometabolic profile across a range of phenotypes of PCOS.

## Methods

The review is reported in accordance with the Preferred Reporting Items for Systematic Reviews and Meta-Analysis (PRISMA) guidelines and was pre-registered in the International Prospective Register of Systematic Reviews (PROSPERO): CRD42018086117. The full protocol is described elsewhere [22].

### Eligibility criteria

Randomised-controlled Trials (RCT), quasi-RCT, and clinical trials were screened according to Population, Intervention, Comparison and Outcome (PICO) criteria: participants were reproductive aged women diagnosed with PCOS according to Rotterdam Criteria 2003 [23], National Institute of Health (NIH) 1990 criteria [24], or Androgen Excess and Polycystic Ovary Syndrome (AE-PCOS) Society 2006 criteria [25]. They were excluded if they were undergoing fertility treatment, taking metformin or OCP, undertaking regular exercise training, or had a diagnosis of any pathology that may be promoting PCOS symptoms.

The intervention could encompass aerobic exercise training, anaerobic exercise training, resistance training, or combinations, of at least two weeks in duration of structured, supervised sessions only. Sessions could be conducted in any setting, as groups or individuals. Crossover trials and interventions that were combined (such as a lifestyle intervention including both exercise and diet management) were excluded. Studies had to include a control group of women with PCOS undertaking no interventions.

Outcomes must have been measured pre-intervention and immediately post-intervention. Primary outcomes identified included low-density lipoprotein cholesterol (LDL-C), high-density lipoprotein cholesterol (HDL-C), total cholesterol (TC), TC:HDL ratio, TG, oxidised LDL, cIMT, fasting blood glucose, HbA1c, blood pressure, waist circumference (WC), waist-to-hip ratio (WHR), abdominal adiposity and any inflammation markers.

Secondary outcomes included total testosterone, free testosterone, sex hormone binding globulin (SHBG), fasting insulin, and homeostatic model assessment for insulin resistance (HOMA-IR).

### Searches

The electronic databases as follows were searched from inception to present: CINAHL Complete (EBSCO), Cochrane Central Register of Controlled Trials (CENTRAL) (Wiley), MEDLINE (EBSCO), Scopus (Elsevier), SPORTDiscus (EBSCO), PEDro (The University of Sydney), PubMed (US National Library of Medicine), ClinicalTrials.gov and UK Clinical Trials Gateway. Only English language publications were sought. Search terms used were PCOS or polycystic ovary syndrome and terms relating to exercise or physical activity interventions. These were adapted for use with all databases; the PubMed search strategy can be found in Online Resource 1.

### Data collection and analysis

#### Study selection

Results from the database searches were imported into RefWorks (ProQuest) and duplicate records were removed. Screening was undertaken in Microsoft Excel (version 16.0). At title and abstract screening phase one reviewer (AW) screened all studies, with a second reviewer screening all in duplicate (MK and DRB).

The full-text of the remaining studies were screened by AW to determine their eligibility for inclusion in the review, with each study checked independently by a second reviewer (MK or DRB). Reasons for exclusion were recorded. Throughout all stages, disagreement between two reviewers was resolved by discussion and input from a third reviewer until a consensus was reached.

### Data extraction

An a priori data extraction form was created in Microsoft Excel (version 16.0). AW extracted all data using the form, with MK and DRB each independently checking all data for consistency. Extracted data included bibliographic information, study characteristics, participant characteristics, intervention and comparison data including adherence and attrition rates, and outcome data including any relevant parameters named in the primary and secondary outcomes. In the case of any missing or unclear data, two attempts were made to contact the corresponding author by email. If no response was received, the missing data was not included in the meta-analysis.

### Risk of bias in individual studies & heterogeneity

The Cochrane Risk of Bias Assessment tool [26] was used to assess quality at the study level as high, low, or unclear risk of bias. The tool evaluates studies based on seven criteria: 1) randomisation generation, 2) allocation concealment, 3) blinding of outcome assessors, 4) blinding patients/study personnel, 5) incomplete outcome data (that is, lost to follow-up), 6) selective outcome reporting, and 7) other risks of bias.

Heterogeneity of results was assessed using the *I*^*2*^ statistic. This statistic was chosen for its simplicity and applicability to meta-analyses regardless of the number of studies involved as described in the literature [27]. It describes the variability, presented as a percentage, in effect estimates that is due to heterogeneity rather than sampling error and is interpreted as follows: 0–40%: might not be important, 30–60%: may represent moderate heterogeneity, 50–90%: may represent substantial heterogeneity, and 75–100%: considerable heterogeneity A result of over 50% was considered significant heterogeneity [28]. Sensitivity analyses were performed as appropriate by removing studies with small sample sizes (<30) or those with a high risk of selection bias.

### Data synthesis

Outcomes measured and presented pre and post intervention were quantitatively synthesised and analysed using RevMan 5 [29]. The *I*^*2*^ statistic, as well as considering clinical and methodological heterogeneity, was used to determine whether random-effects or fixed-effects meta-analysis was used. Forest plots were generated where a *P* value of <0.05 was considered statistically significant. Each outcome for each study was recorded with mean and standard deviation (SD) of each group, effect size (difference between means), 95% confidence intervals (CI), and study weighting. Pooled mean difference, 95% CI, *P*-values and *I*^*2*^ statistic were also recorded for each outcome.

### Confidence in findings

The Grading of Recommendations Assessment, Development and Evaluation (GRADE) tool was used to grade the quality of the evidence and the strength of each finding [30]. GRADE uses a scoring system (very low, low, moderate, high) to grade each finding in several areas including limitations, consistency, directness, and publication bias. The use of a consistent and transparent approach to evaluating recommendations increases the facilitation of critical appraisal and improves communication of these judgments [30].

## Results

### Results of the search

**Fig. 1:**
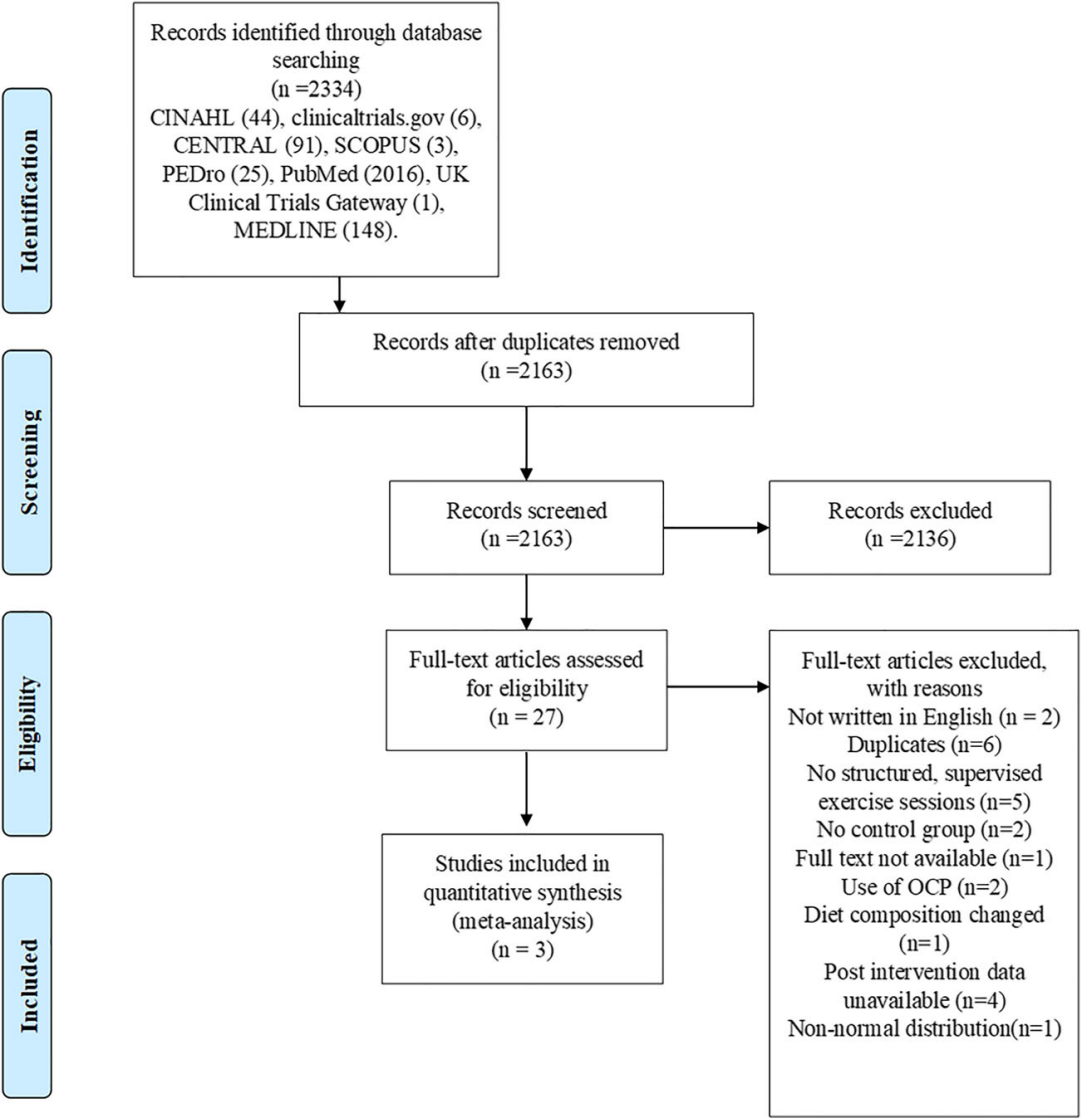
**PRISMA flow-chart**From: Moher D, Liberati A, Tetzlaff J, Altman DG, The PRISMA Group (2009). Preferred Reporting Items for Systematic Reviews and Meta-Analyses: The PRISMA Statement. PLoS Med 6(7): e1000097. 10.1371/journal.pmed100009

The initial search of databases identified a combined total of 2,334 records. Once duplicates were removed, 2,163 records remained for title and abstract screening. Records were excluded (*n* = 2,136) because the title and abstract screening revealed that the articles did not meet the inclusion criteria. Twenty-seven articles were selected for full-text eligibility screening. Twenty-four were excluded for the reasons identified in Fig. 1.

### Study design and data handling

Three studies were included in the meta-analysis. One was an exercise only RCT [31] and two were exercise only non-randomised clinical trials [32, 33] All compared an exercise intervention to a control group or standard care.

Two studies presented data as mean and SD [31, 32], and one presented data as mean and 95% CI [33]. Data from the latter study were converted into mean and SD. Data were converted into the most common unit used for each variable if there were discrepancies.

Sensitivity analysis was performed either by removing studies with small sample sizes (<30 participants) from the pooled data or by removing those with a high risk of selection bias.

### Participant characteristics

Table 1 is a summary of characteristics of the three included studies. Across all studies, there was a total of 231 participants, with 117 receiving an exercise intervention and 114 controls. Total participants ranged from 124 [32] to 17 [33]. The 2003 Rotterdam criteria was used to reach a PCOS diagnosis in all three studies [31–33]. The mean age of participants was 26 years, ranging from 22 [31] to 28 years [33].

**Table 1:** Characteristic of included studies

**Study**	**Type**	**Diagnosis**	**Exercisers**	**Controls**	**Duration**	**Frequency**	**Session Length**	**Mode**	**Intensity**	**Outcomes Reported**	**Significant Improvement Between Groups**^**α**^
Giallauria et al. 2008 [30]	CT	Rotterdam	*N* = 62 BMI = 29.2 kg/m^2^	*N* = 62 BMI = 29.5 kg/m^2^	3 months	3/week	30 min	Bicycle ergometer	60–70% of VO_2_ max	LDL-C, HDL-C, TC, TG, Fasting Glucose, WHR, TT, SHBG, CRP, SBP, DBP	WHR* and CRP*
Sprung et al. 2013 [32]	CT	Rotterdam	*N* = 10 BMI = 31 kg/m^2^	*N* = 7 BMI = 35 kg/m^2^	16 weeks	3/week for 11 weeks 5/week for 5 weeks	30 min for 11 weeks, 45 min for 5 weeks	Participant preference	30% HRR for 11 weeks, 60% HRR for 5 weeks	LDL-C, HDL-C, TC, TG, Fasting Glucose, WC, TT, SHBG. HOMA-IR	TC** and LDL-C**
Vigorito et al. 2007 [31]	RCT	Rotterdam	*N* = 45 BMI = 29.3 kg/m^2^	*N* = 45 BMI = 29.4 kg/m^2^	3 months	3/week	30 min	Bicycle ergometer	60–70% VO_2_ max	LDL-C, HDL-C, TC, TG, Fasting Glucose, WC, WHR, TT, HOMA-IR, SBP, DBP, CRP	WC, WHR

Study is lead author and year of publication. Type: CT = controlled trial, RCT = randomised controlled trial. Diagnosis refers to the specific criteria that the researchers used to confirm PCOS diagnosis: Rotterdam = European Society for Human Reproductive and Embryology/American Society for Reproductive Medicine (2003). N = number of participants randomised into each arm of the study. BMI = mean body mass index (kg/m^2^) of participants in each arm at study entry. Duration, frequency, session length, mode and intensity refer to intervention characteristics. HRR = heart rate reserve, VO_2_ max = maximum oxygen update, LDL-C = low-density lipoprotein cholesterol, HDL-C = high-density lipoprotein cholesterol, TC = total cholesterol, TG = triglycerides, WC = waist circumference, WHR = waist-to-hip ratio, TT = total testosterone, SHBG = sex hormone-binding globulin, HOMA-IR = homeostatic assessment of insulin resistance, SBP = systolic blood pressure, DBP = diastolic blood pressure, CRP = C-reactive protein. α = statistically significant.

### Intervention characteristics

The exercise intervention duration in two studies was three months [31, 32], and one was 16 weeks [33]. All of the studies had an exercise frequency of three times per week [31–33]. One study began with three sessions per week for 11 weeks and then progressed to five sessions per week for five weeks [33]. Exercise intensity was determined by a percentage of VO_2_max [31, 32] or heart rate reserve (HRR) [33]. All were aerobic exercise interventions. Session length was 30 min in all three studies [31–33] increasing to 45 min after 11 weeks in one [33]. Two studies were performed on a bicycle ergometer [31, 32], and one was performed on a stationary cycle, treadmill or elliptical machine according to participant preference [33].

All three studies reported that all participants completed the study protocol [31–33]. All studies reported a mean adherence of ≥80%. All studies included women of reproductive age with a confirmed PCOS diagnosis. All studies specifically mentioned exclusion of participants who were taking OCP, metformin, or other hormonal, anti-androgen or carbohydrate metabolism modification drugs. All studies also specifically mentioned the exclusion of other conditions that could promote hyperandrogenism, such as Cushing’s Syndrome and congenital adrenal hyperplasia. All studies excluded those with thyroid dysfunction, diabetes, cardiovascular disease or other renal or hepatic diseases. Only one study confirmed exclusion of smokers and the exclusion or participants who undertook regular exercise [33]. Two studies did not specify a formal sample size calculation [31, 32] and another based this on an outcome of flow-mediated dilation [33].

### Risk of bias in included studies

**Fig. 2:**
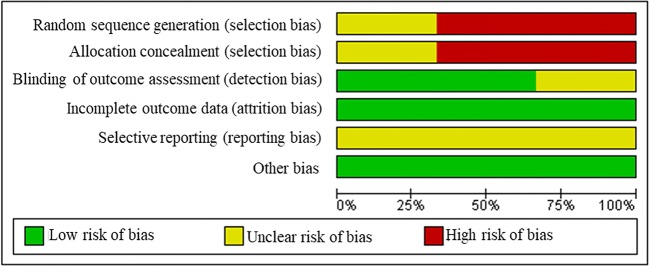
**Risk of bias graph**. Review authors’ judgements about each risk of bias item presented as percentages across all included studies

**Fig. 3:**
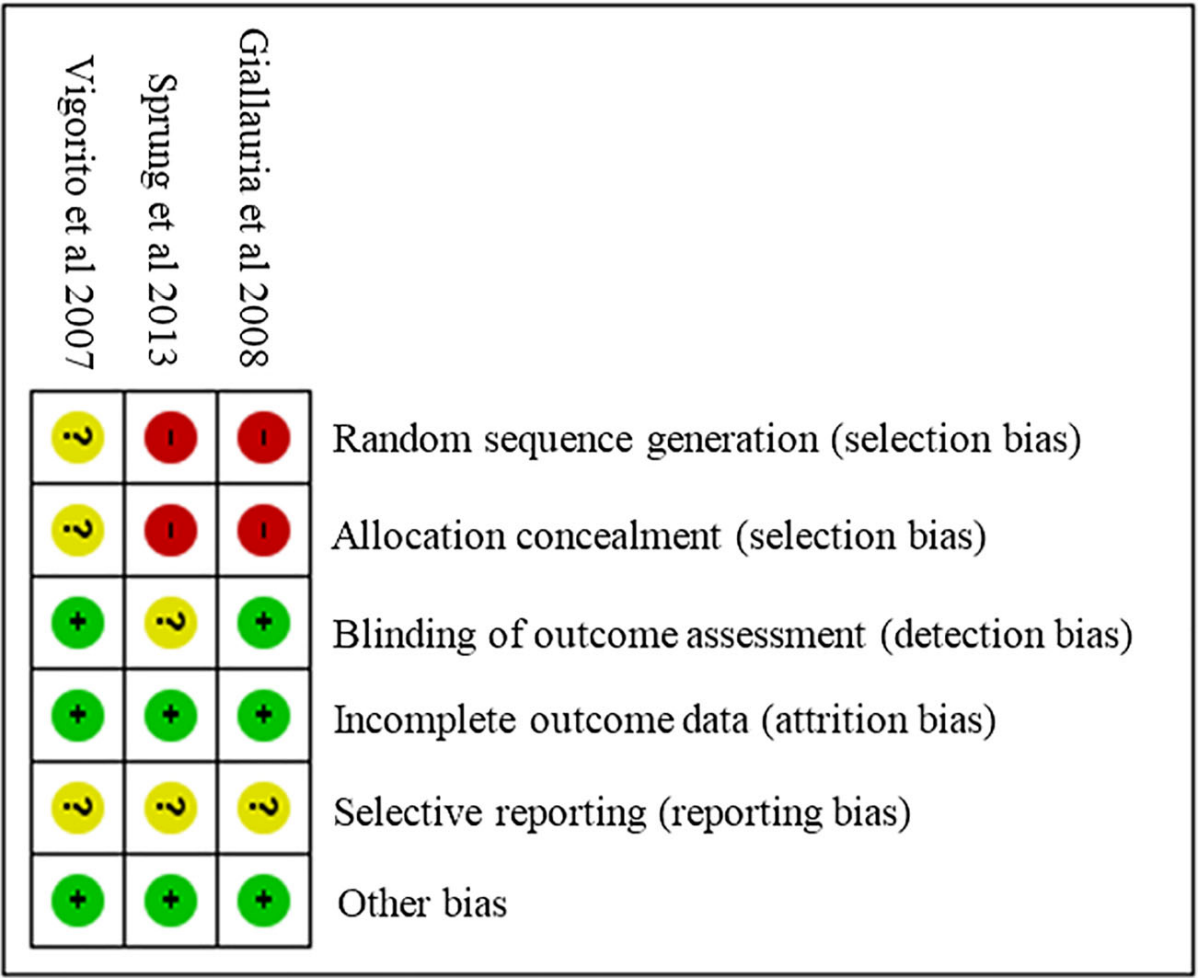
**Risk of bias summary**. Review authors’ judgements about each risk of bias item for each included study

The authors’ judgements about each risk of bias category are presented as percentages across all included studies in Fig. 2. A summary of the authors’ judgements of each risk of bias item for each included study are presented in Fig. 3. Further information outlining how each judgement was reached for each category in each included study is available in Online Resource 2.

Two studies (66.6%) were judged to have a high risk of selection bias because participants were allocated to groups based on their own choice [32, 33] and one (33.3%) was judged to have an unclear risk of selection bias because the authors did not report a method for randomisation or allocation concealment [31].

Performance bias was excluded from the assessment as all the studies included supervised exercise sessions so it is impossible to blind participants to this type of intervention while promoting exercise behaviour. Two studies (66.6%) were judged to have a low risk of detection bias because the blinding of outcome assessment was ensured, or the outcome measurement was not likely to be influenced by lack of blinding [31, 32]. The remaining study was judged to have an unclear risk of detection bias because the authors did not address this outcome. All studies were judged to have a low risk of attrition bias either due to zero reported attrition rate, and all were judged to have an unclear risk of reporting bias because prospective protocols could not be located [31–33]. Additionally, we assessed whether of adherence (reported as <80%) may have presented a high risk of ‘other sources of bias’, and all were judged to be at a low risk [31–33].

### Reporting of outcomes

All three studies reported on outcomes relating to lipid profile (such as HDL-C, LDL-C, TC and TG) but no studies reported oxidised LDL. All studies included either WC or WHR. Two studies reported fasting blood glucose and HOMA-IR measures [31, 33], and one reported just fasting blood glucose [32]. On androgen profile, all three studies reported total testosterone [31–33] and two reported sex hormone binding globulin (SHBG) in addition [32, 33]. Two studies reported systolic blood pressure (SBP) and diastolic blood pressure (DBP) [31, 32]. Only two studies reported inflammation markers and both of those reported C-reactive protein (CRP) [31, 32].

### Effects of exercise versus control

Following our study inclusion criteria, only three studies could be included in the meta-analysis. As such, subgroup analyses of exercise intensity, type and frequency were not performed. Subgroup analysis for intervention duration may have been possible, but given there would be two studies in one category and one in the other, it was deemed to be uninformative and potentially misleading [34]. Effect estimates, 95% CI and *I*^2^ values are listed in Table 2 for each outcome.

**Table 2:** Mean difference, 95% CI, P and *I*^2^ value for each outcome analysed

**Outcome**	**Studies**	**N**	**MD**	**Lower**	**Upper**	**P**	**I**^**2**^**(%)**
HDL-C (mg/dL)	3	231	−2.97	−6.62	0.68	0.11	0
LDL-C (mg/dL)	3	231	−4.10	−13.32	5.22	0.39	42
TC (mg/dL)	3	231	−4.78	−9.24	−0.32	0.04	14
TG (mg/dL)	2	214	1.55	−4.66	7.76	0.63	0
Fasting Glucose (mg/dL)	2	214	−1.75	−3.46	−0.04	0.04	0
WC (cm)	2	107	−1.97	−3.35	−0.59	0.005	0
WHR	2	214	−0.05	−0.08	−0.02	0.0003	0
TT (nmol/L)	3	231	−0.20	−0.38	−0.02	0.03	47
SHBG (nmol/L)	2	141	4.05	1.79	6.31	0.0004	0
CRP (mg/L)	2	214	−0.34	−0.54	−0.15	0.0006	0
SBP (rest) (mmHg)	2	214	−4.40	−7.13	−1.66	0.002	0
DBP (rest) (mmHg)	2	214	−0.80	−1.96	0.37	0.18	0

*N* number of participants; *MD* Mean difference; *LDL-C* low-density lipoprotein cholesterol; *HDL-C* high-density lipoprotein cholesterol; *TC* total cholesterol; *TG* triglycerides; *WC* waist circumference; *WHR* waist-to-hip ratio; *TT* total testosterone; *SHBG* sex hormone-binding globulin; *HOMA-IR* homeostatic assessment of insulin resistance; *SBP* systolic blood pressure; *DBP* diastolic blood pressure; *CRP* C-reactive protein

Additionally, free testosterone measures were not available. Total testosterone measures indicated that the mean values for participants (231) in the studies eligible for meta-analysis were hyper-androgenemic, based on total testosterone (TT) concentrations of >2.0 nmol/L [35–37], therefore subgroup-analysis of androgen profile could not be conducted.

#### Primary outcomes

##### Blood lipids

All three studies (231 participants) in the meta-analysis assessed changes in LDL-C, HDL-C, TC and TG (231 participants). We observed no effect of exercise versus control on LDL-C, HDL-C or TG. We found a statistical effect of exercise on TC versus control (−4.70 mg/dl, 95% CI -9.24, −0.32, I^2^ = 14%). When the study with a small sample size was removed [33], the effect was no longer statistically significant.

Of the three studies in the analysis, one reported a significant decrease in LDL-C (−0.7 mmol/L, 95% CI -1.1 to −0.3, *P* = 0.001) and TC (−0.20 mmol/L, 95% CI -0.28 to −0.04, *P* = 0.01) when compared to the control group [33].

##### Fasting blood glucose

Data from the three studies (231 participants) pooled in the meta-analysis showed a significant favourable effect of exercise on fasting glucose concentrations versus controls (−1.75 mg/dL, 95% CI -3.45, −0.5, I^2^ = 0%). When the study with a small sample size was removed [33], the effect remained significant (−1.75 mg/dL, 95% CI -3.46, −0.4, 214 participants, I^2^ = 0%).

##### Measures of abdominal obesity

Two studies (107 participants) were pooled in the meta-analysis to assess changes to WC and WHR. A statistical favourable effect of exercise on WC (−1.97 cm, 95% CI -3.35, −0.59, I^2^ = 0%) and a small but statistical favourable effect of exercise on WHR (−0.05, 95% CI -0.09, −0.01, I^2^ = 0%) compared to the control group was observed.

One study reported a significant decrease in WC (*P* < 0.01) and WHR (*P* < 0.05) in the exercise group when compared to the control group [31]. One other reported significant decreases in WHR (P < 0.05) in the exercise group compared to control [32].

##### Blood pressure

Two studies (214 participants) were pooled in the meta-analysis to assess changes in SBP and DBP at rest. The results indicated a statistical favourable effect of exercise on SBP in comparison to controls (−4.40 mmHg, 95% CI -7.13, −1.66, I^2^ = 0%) but no effect was observed for DBP.

Of the two studies reporting SBP and DBP, one did not note any statistical effect of exercise on SBP or DBP in comparison to controls [32]. The other study [31] reported a significant (P < 0.01) decrease in SBP after the exercise intervention, but this was not significant in comparison to the control group.

##### C-reactive protein

Two studies (114 participants) included in the meta-analysis recorded changes in CRP. The authors observed a small but statistical favourable effect of exercise on CRP compared to controls (−0.34 mg/l, 95% CI -0.54, −0.14, I^2^ = 0%). Both studies had a sample size >30.

Of the two studies one reported significant improvement after exercise only [31] and the other found significant improvement after exercise and between-groups [32]. Both studies were ≥ 12 weeks in duration, with sessions of 30 min on a bicycle ergometer.

#### Secondary outcomes

##### Total Testosterone and sex hormone binding globulin

Three studies (231 participants) were pooled to assess changes in TT. The authors found a significant favourable effect of exercise on TT compared to controls, although moderate heterogeneity was noted (−0.20 nmol/l, 95% CI -0.38, −0.02, I^2^ = 47%). Removal of the study with a small sample size [33] mitigated I^2^ to 35% and increased the statistical effect estimate (−0.24 nmol/l, 95% CI -0.43, −0.05, 114 participants). The same result was also observed when removing the study with the highest risk of bias [33].

Only two of the studies reporting TT also reported changes to SHBG (114 participants). The meta-analysis indicated a favourable effect of exercise on SHBG concentrations (4.10, 95% CI 1.79, 6.31, I^2^ = 0%). However, of note, both studies had a high risk of bias in two domains.

##### Homeostatic model assessment of insulin resistance

Only one study eligible for meta-analysis reported HOMA-IR and as such pooled analysis could not be conducted. No studies reported any significant improvement in HOMA-IR after exercise.

Figures 4, 5, 6, 7, 8, 9, 10, 11, 12, 13, 14 and 15 show the comparisons for each outcome and subsequent forest plot.

**Fig. 4:**
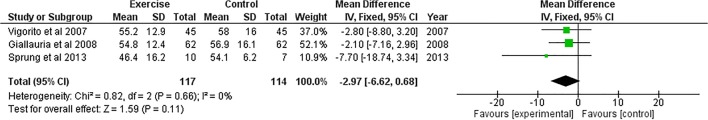
Forest plot of comparison: 1 – all interventions, outcome: 1.1 – HDL-C (mg/dL)

**Fig. 5:**
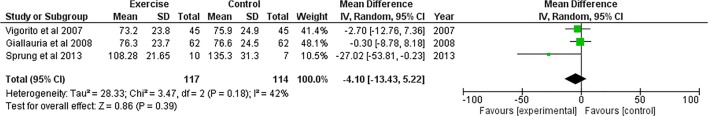
Forest plot of comparison: 1 – all interventions, outcome: 1.1 – LDL-C (mg/dL)

**Fig. 6:**
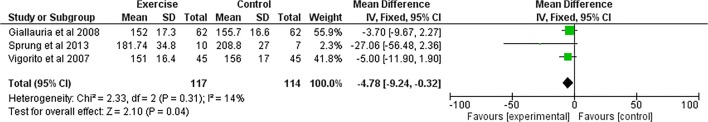
Forest plot of comparison: 1 – all interventions, outcome: 1.3 – TC (mg/dL)

**Fig. 7:**
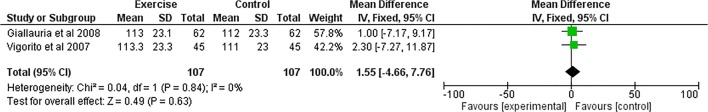
Forest plot of comparison: 1 – all interventions, outcome: 1.4 – TG (mg/dL)

**Fig. 8:**
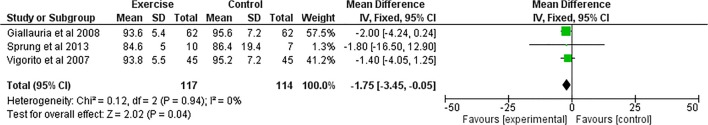
Forest plot of comparison: 1 – all interventions, outcome: 1.5 – Fasting blood glucose (mg/dL)

**Fig. 9:**
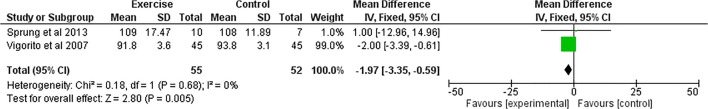
Forest plot of comparison: 1 – all interventions, outcome: 1.6 – Waist circumference (cm)

**Fig. 10:**
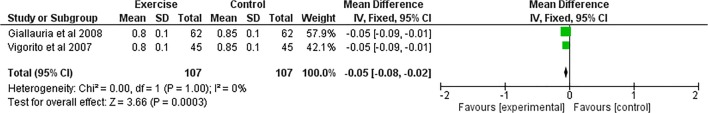
Forest plot of comparison: 1 – all interventions, outcome: 1.7 – Waist-to-hip ratio

**Fig. 11:**
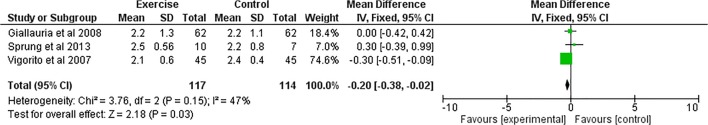
Forest plot of comparison: 1 – all interventions, outcome: 1.8 – Total testosterone (nmol/L)

**Fig. 12:**
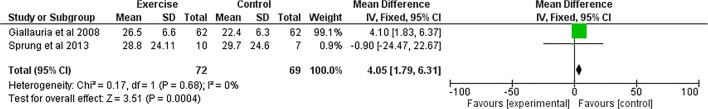
Forest plot of comparison: 1 – all interventions, outcome: 1.9 – Sex hormone-binding globulin (nmol/L)

**Fig. 13:**
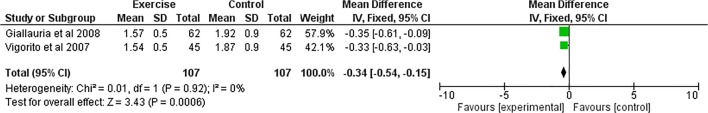
Forest plot of comparison: 1 – all interventions, outcome: 1.10 – C-reactive protein (mg/L)

**Fig. 14:**
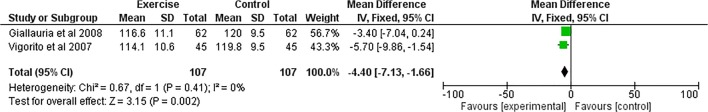
Forest plot of comparison: 1 – all interventions, outcome: 1.11 – Systolic blood pressure (rest) (mmHg)

**Fig. 15:**
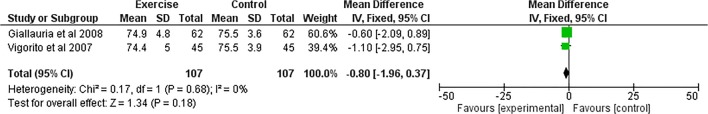
Forest plot of comparison: 1 – all interventions, outcome: 1.12 – Diastolic blood pressure (rest) (mmHg)

### Quality of the evidence

Using GRADE, Table 3 provides an evidence profile to reflect the extent of confidence that each estimate of effect from the pooled data-analysis is correct. Evidence has been downgraded for all outcomes due to the presence of serious study design limitations, including small sample size (≤30 participants), unclear or inappropriate randomisation or allocation procedures and non-randomised controlled trials. Subsequently, all evidence could only begin at a maximum of moderate quality.

**Table 3:** GRADE evidence profile to assess confidence in effect estimates for each outcome

**No of Studies (No. of participants)**	**Quality Assessment**					**Summary of Findings**		
	Study Limitations*	**Consistency**	**Directness**	**Precision**	**Publication Bias**	**P Value**	**Effect(95% CI)**	**Quality**
**HDL-C**								
3 (231)	Serious limitations (−1)	No important inconsistency	Direct	Imprecision (−1)^a^	Unlikely	0.11	−2.97 (−6.62, 0.68)	++, Low
**LDL-C**								
3 (231)	Serious limitations (−1)	No important inconsistency	Direct	Imprecision (−1)^a^	Unlikely	0.39	−4.10 (−13.43, 5.22)	++, Low
**TC**								
3 (231)	Serious limitations (−1)	No important inconsistency	Direct	No important imprecision	Unlikely	0.04	−4.78 (−9.24, −0.32)	+++, Moderate
**WC**								
2 (107)	Serious limitations (−1)	No important inconsistency	Direct	No important imprecision	Unlikely	0.005	−1.97 (−3.35, −0.59)	+++, Moderate
**WHR**								
2 (107)	Serious limitations (−1)	No important inconsistency	Direct	No important imprecision	Unlikely	0.0003	−0.05 (−0.08, −0.02)	+++, Moderate
**Fasting Glucose**								
2 (214)	Serious limitations (−1)	No important inconsistency	Direct	No important imprecision	Unlikely	0.04	−1.75 (−3.46, −0.04)	+++, Moderate
**TT**								
3 (231)	Serious limitations (−1)	Moderate Heterogeneity (−1)^b^	Direct	No important imprecision	Unlikely	0.03	−0.20 (−0.38, −0.02)	++, Low
**SHBG**								
2 (141)	Serious limitations (−1)	No important inconsistency	Direct	No important imprecision	Unlikely	0.0004	4.05 (1.79, 6.31)	+++, Moderate
**CRP**								
2 (214)	Serious limitations (−1)	No important inconsistency	Direct	No important imprecision	Unlikely	0.0006	−0.34 (−0.54, −0.15)	+++, Moderate
**SBP**								
2 (107)	Serious limitations (−1)	No important inconsistency	Direct	No important imprecision	Unlikely	0.002	−4.40 (−7.13, −1.66)	+++, Moderate
**DBP**								
2 (107)	Serious limitations (−1)	No important inconsistency	Direct	Imprecision (−1)^a^	Unlikely	0.18	−0.80 (−1.96, 0.37)	++, Low

*LDL-C* low-density lipoprotein cholesterol; *HDL-C* high-density lipoprotein cholesterol; *TC* total cholesterol; *TG* triglycerides; *WC* waist circumference; *WHR* waist-to-hip ratio; *TT* total testosterone; *SHBG* sex hormone-binding globulin; *HOMA-IR* homeostatic assessment of insulin resistance; *SBP* systolic blood pressure; *DBP* diastolic blood pressure; *CRP* C-reactive protein

*unclear randomisation and allocation, non-randomised controlled trials, small sample size (<30).

^a^ confidence interval includes possible benefit in both directions.

^b^ I^2^ 47%.

Moderate heterogeneity was observed for only one outcome. Also, there was no important inconsistency of mean post-intervention values in most of the analyses. No outcomes were downgraded for indirectness, because all studies directly compared an exercise intervention versus usual care or control, with explicit exclusions of confounding medications. Where CI were wide or indicated possible benefit in both directions, evidence was downgraded due to imprecision and uncertainty of results. Publication bias of all outcomes was considered unlikely, since the authors conducted a thorough and comprehensive search of relevant databases, and no studies eligible for analysis declared any conflict of interest or funding sources that may have influenced publication.

## Discussion

This systematic review and meta-analysis identified three studies, including 231 participants with PCOS, that isolated and examined the effect of structured, supervised exercise on cardiometabolic outcomes in PCOS. Various recently published reviews have examined the effects of exercise and/or lifestyle modification on facets of PCOS [17, 38–40]. To the authors’ knowledge, this is the only recent review that has aimed to isolate the effects of exercise alone in comparison with control/standard care, without the inclusion of dietary, pharmacological or behavioural modification programmes.

### Summary of main findings

Analysis of pooled data indicated, in the comparison of exercise and control, statistical favourable effects of exercise on TC, fasting glucose, WC, WHR, SBP, CRP, TT and SHBG using post-intervention scores. This supports the role of exercise as a treatment in the improvement of several cardiovascular risk factors in PCOS, including abdominal adiposity, insulin sensitivity, endothelial dysfunction and androgen profile.

### Primary outcomes

The authors found a statistically-significant effect of exercise was observed on TC versus control (−4.70 mg/dl, 95% CI -9.24, −0.32, I^2^ = 14%), *P* = 0.04, but meta-analysis revealed no other significant changes to lipid profile in PCOS women. Other reviews have produced inconsistent results; a comprehensive, qualitative review [17] mostly found no significant effects of exercise only (without a dietary component) on lipid profile in PCOS, and those studies reporting significant improvements in TC involved a combined dietary and exercise component. Conversely, a recent review [38] noted a statistical effect of exercise on TC concentrations in PCOS in a pooled meta-analysis of just two studies (−0.09 mmol/L, 95% CI -0.10, −0.07), though it is not clear if this was based on exercise versus control only. Subsequently, sensitivity analysis rendered the pooled effect estimate non-significant. Additionally, since TC is the sum of LDL-C and HDL-C, the clinical relevance of this measure may be misleading, since LDL-C and HDL-C have contrasting roles within the vascular system and a change to either would affect the measure of TC [41]. TC:HDL appears to be a better predictor of cardiovascular risk than TC or LDL-C [42, 43].

Despite these results, exercise has been shown to have a positive effect on HDL-C and TG in healthy populations and those presenting with metabolic syndrome [44–46] with the latter sharing some cardiovascular risk factors with PCOS. This discrepancy may be due to the intervention characteristics shared by the three included studies (3/week, 30 min-session). It has been reported that that changes to HDL-C and TG are more likely with an energy expenditure of 1200 kcal/week [45]; these interventions may be unlikely to produce this output at lower intensities. Additionally, a 2004 review [44] indicates that interventions should be longer in duration (>20 weeks) to induce positive changes to HDL-C and TG in people with metabolic syndrome.

Pooled analysis of post-intervention values indicated a significant effect estimate of exercise versus control on fasting glucose concentrations (−1.75 mg/dl, 95% CI -3.45, −0.5, I^2^ = 0%), *P* = 0.04. This effect remained significant after sensitivity analysis. This finding is in line with a recent review that indicated a statistically significant effect estimate of lifestyle modification versus minimal intervention on fasting blood glucose in PCOS (−2.3 mg/dL, 95% CI, −4.5 to −0.1, I^2^ = 72%) P = 0.04 [39]. However, statistical heterogeneity was noted, and exercise and dietary/behavioural modification were combined under ‘lifestyle intervention’. Two other reviews [38, 40] found no significant effects of lifestyle or exercise interventions on fasting blood glucose in PCOS. Despite this, various studies have demonstrated that aerobic exercise training enhances glucose disposal rate in women with PCOS [12, 47]. The mean fasting blood glucose range for the three studies in the pooled analysis was 84.6–95.6 mg/dL, which are all considered to be in the normal range of <100 mg/dL [48]. This is not unusual, because women with PCOS can maintain normal fasting glucose at the expense of increased insulin secretion [36]. Nevertheless, it is difficult to assess the clinical relevance of this outcome without comparative data on insulin sensitivity.

We noted a statistically-favourable effect of exercise versus control on WC (−1.97 cm, 95% CI -3.35, −0.59, I^2^ = 0%), *P* = 0.005, and WHR (−0.05, 95% CI -0.09, −0.01, I^2^ = 0), *P* = 0.003, in two studies. This is in agreement with two other reviews [38, 40], although one combined exercise and dietary modification under lifestyle intervention [40]. WC and WHR have been shown to be, in some cases, a better indicator of health risk than BMI [49] because they measure abdominal obesity, a condition strongly associated with cardiovascular risk factors [50]. A decrease in WC and WHR has also been associated with improvements in glucose metabolism [51].

The authors observed that exercise had a statistically-significant effect on SBP in comparison to control (4.40 mmHg, 95% CI -7.13, −1.66, I^2^ = 0%), *P* = 0.0003. This has been observed after lifestyle intervention in PCOS in another review (−5.01 mmHg, 95% CI -6.63, −3.39, *P* < 0.05, I^2^ = 0%) [38]. A meta-analysis of RCTs [52] has indicated that aerobic exercise training produces a small but statistical improvement in blood pressure, even in the absence of weight loss, in normotensive adults. Blood pressure values among this population have been inversely associated with insulin sensitivity [44]. The mean data from the meta-analysis indicates that participants were normotensive (≤120 mmHg). We observed improvements of WC and WHR, shown to be associated with insulin sensitivity in PCOS [53]. As such, improvement in insulin regulation is a plausible explanation for several of the observed effect estimates.

We observed a favourable statistical effect of exercise on CRP versus control (−0.34 mg/l, 95% CI -0.54, −0.14, I^2^ = 0%) *P* < 0.001. This finding is in agreement with another review that found favourable effects of lifestyle modification versus usual care (−0.47 mmol/L, 95% CI -0.80, −0.15, *P* = 0.004, I^2^ = 0%). Indeed, PCOS has been linked to an inflammatory state characterised by increased levels of CRP [19, 54]. However, the clinical relevance of this finding may be tenuous; the mean CRP range for the studies in the pooled analysis was 1.54–1.92 mg/L, which are considered to be within the normal range [55] and as such this may not indicate an inflammatory state in the participants. Also, the effect may not be reproduced in populations with a higher than normal value.

### Secondary outcomes

Pooled data analysis indicated a statistical favourable effect of exercise versus control on TT(−0.20 nmol/L, 95% CI -0.38, −0.02, I^2^ = 47%) *P* = 0.03, and SHBG (4.05, 95% CI 1.79, 6.31, I^2^ = 0%) P < 0.001. Both outcomes were derived from at least one study with a high risk of bias for randomisation and allocation procedures. Nevertheless, a previous review has noted a statistical lowering of fasting insulin levels in the exercise group compared to the control group in PCOS (−0.95 μU/mL, 95% CI -1.48, −0.43, *P* < 0.05, I^2^ = 0%) [38]. Additionally, a qualitative systematic review found evidence for improved insulin sensitivity following exercise in PCOS [17]. An improvement in insulin sensitivity following exercise could therefore be an explanation for both reduced TT and increased SHBG; hyperinsulinemia causes an increase in free androgen plasmatic levels both through the stimulation of ovarian androgen synthesis, and by suppressing hepatic production of SHBG [56]. We were not able to perform a meta-analysis on free testosterone; caution is advised when measuring TT alone, because women with PCOS can have TT in the normal range but have high concentrations of free and bioavailable testosterone due to lower concentrations of SHBG [36]. However, the data indicate that the participants in the meta-analysis had low enough SHBG concentrations (<30 nmol/L), even post-intervention, to indicate hyperandrogenism [36]. This provides further plausibility to the explanation that exercise may have mitigated insulin hypersecretion, thereby increasing hepatic production of SHBG and reducing ovarian androgen synthesis to the effect of reduced TT.

### Overall completeness and applicability of evidence

One study in the analysis was an RCT and two were non-RCT. This limits the overall applicability of the evidence, particularly where participants were allocated to groups based on preference. Although the studies specified no statistical differences in baseline characteristics, it is possible that the adherence and attrition rates are not truly reflective of those that would be observed in gold-standard RCTs.

Only one study specified formal sample size calculations, and this study had a small sample size (17 participants). In samples of this size, variance in scores is likely to affect statistical significance and applicability to the general PCOS population is limited.

Sub-group analysis based on androgen profile was not possible, because the studies included in the meta-analysis indicated that the mean TT concentration for all participants were high enough to constitute hyperandrogenemia. Typical cut-off values of TT for hyperandrogenemia are generally >2.1 nmol/L [36, 37] and post-intervention values for all participants in the meta-analysis (*n* = 231) ranged from 2.1–2.5 nmol/L. The results of the meta-analysis may therefore have limited applicability to normo-androgenic phenotypes and differences in treatment responsiveness between phenotypes have not been highlighted.

An important characteristic of the review was to only include trials where OCP was clearly excluded. The authors wanted to avoid the contamination of the data by the hormonal and metabolic changes associated with the OCP, particularly those with low or anti-androgenic properties, such as hepatic synthesis of SHBG that reduces free testosterone concentrations [57]. Additionally, in overweight or obese women with PCOS, research suggests that certain types of OCP containing desogestral or cyproterone acetate can aggravate insulin resistance and decrease glucose tolerance [58–60]. Because of the considerable variability in the presentation of clinical and metabolic symptoms of PCOS, including varying levels of glucose tolerance, hyperandrogenism and insulin sensitivity, as well as the variation in the types and metabolic effects of OCPs used to manage PCOS symptoms, we excluded those participants taking OCP to reduce the effects of inter-person variability in the meta-analysis.

PCOS is the most common cause of infertility [61]. It is estimated that 40% of women with PCOS are affected by infertility or difficulty conceiving [62]. As a result, approximately up to 95% of anovulatory women seeking or receiving fertility treatment have PCOS [45]. Therefore, although OCP may be a front-line management tool in PCOS in women not aiming to conceive [58], there exists a substantial proportion of women with PCOS that are not taking OCP, many of whom are encouraged to improve their health to increase chances of conception, indicating that the findings of this review have applicability to this subset of the population.

### Potential biases in the review process and limitations

We restricted our eligibility criteria to articles published in the English language. Consequently, it is possible that additional information from trials that would have otherwise met the inclusion criteria may have been excluded. Also, trials were only eligible for inclusion if the full-text could be obtained; subsequently at least one eligible trial could not be included because the abstract was for a conference and the full-text had not been published. These factors may contribute to publication bias. Due to a lack of trials in the meta-analysis, funnel plots could not be utilised for the analysis of publication bias.

Some difficulty in study selection occurred due to a lack of trials that explicitly excluded the use of OCP and other hormonal or metabolism-altering drugs. The authors could only select studies if this was specifically excluded, and as such some studies may have been excluded for not providing such a statement. Similarly, at least one gold-standard RCT was excluded due to the use of non-normally distributed data and non-parametric tests. These data could have influenced findings if they could be synthesised for meta-analysis and thus had to be excluded.

Many of the outcomes were based on studies with serious limitations, including a high risk of selection bias, and small magnitude effect estimates. This limits the quality of the evidence, despite the directness and consistency of the evidence for most outcomes. As noted, the generalisability may also be limited by the high occurrence and selection bias, and particularly by study designs which allowed participant allocation based on preference rather than true randomisation.

### Future research recommendations

Most studies featured moderate-intensity aerobic interventions, with less emphasis on resistance training in the literature, therefore different types of exercise intervention could not be compared. Current physical activity guidelines recommend that adults undertake activity to improve muscle strength on at least two days a week [63]. As such, a greater emphasis should be placed on the inclusion of resistance exercises in exercise interventions to identify additional benefits to cardiometabolic health in PCOS. Future consideration could also be given to tools for self-reporting physical activity, such as the Global Physical Activity Questionnaire, as well as interventional studies.

## Conclusions

The results of the pooled data analysis indicated that moderate aerobic exercise interventions ≥3 months in duration, with a frequency of 3/week for at least 30-min-long sessions, may have favourable effects on various cardiometabolic risk factors including TC, fasting blood glucose, WC, WHR, SBP and CRP in women with PCOS. Additionally, we observed that if participants have TT and SHBG concentrations outside of normal ranges, this type of intervention could improve androgen profile in comparison to usual care.

As indicated by our analysis of the quality of the evidence, various outcomes were judged to be of a moderate quality, with statistically significant, precise effect estimates. Nonetheless, results should be interpreted with caution due to the presence of serious methodological limitations including a lack of gold-standard RCTs and a high risk of selection bias.

We conducted a thorough search of nine databases from inception to present but were only able to find three eligible studies that isolated the effects of exercise alone versus usual care that explicitly excluded the use of OCP and other hormonal or metabolism-altering drugs. Only one of these was a gold-standard RCT, albeit judged to have an unclear risk of selection bias due to unclear randomisation or allocation procedures. This review highlights the limitations of the available literature. More gold-standard RCTs that can make direct comparisons between treatment options for PCOS, including exercise, pharmacological, behavioural and dietary interventions could provide greater precision for future recommendations of treatment options, including the efficacy of exercise in comparison to other treatments. However, the authors acknowledge that this may have limited applicability to the general population; often, patients with PCOS may undertake combined interventions to get the best results, and studies designed in this manner may provide greater applicability in that regard.

## Electronic supplementary material

Online Resource 1 PubMed Search Strategy. (PDF 72 kb)

Online Resource 2 How each judgement was reached for each category in each included study. (PDF 89 kb)
