# Supplementary material for: The effects of physical exercise on cardiometabolic outcomes in women with polycystic ovary syndrome not taking the oral contraceptive pill: a systematic review and meta-analysis
Source: J Diabetes Metab Disord. 2019 Jul 29;18(2):597–612. doi: 10.1007/s40200-019-00425-y (PMC6915192; doi:10.1007/s40200-019-00425-y)
Supplement: Supplementary file 1 — PubMed Search Strategy. (PDF 72 kb) [file 40200_2019_425_MOESM1_ESM.pdf]

The effects of exercise on cardiometabolic outcomes in women with polycystic ovary syndrome not taking the oral contraceptive pill: A systematic review and meta-analysis.

Journal of Diabetes and Metabolic Disorders

Ms Amie Woodward, BSc (Hons), MSc. Doctoral Researcher, Faculty of Health and Wellbeing, Sheffield Hallam University, Sheffield, S10 2BP. Amie.woodward@shu.ac.uk*

Dr David Broom, PhD. Reader of Physical Activity and Health, Faculty of Health and Wellbeing, Sheffield Hallam University, Collegiate Crescent, Sheffield, S10 2BP. D.r.broom@shu.ac.uk

Deborah Harrop, BA (Hons), MA, PGCERT. Information Scientist, Nursing and Midwifery, Faculty of Health and Wellbeing, Sheffield Hallam University. D.harrop@shu.ac.uk

Dr Ian Lahart, BA (Hons), MSc, PGCERT, PhD. Senior Lecturer in Sport and Exercise Physiology. Institute of Human Science, University of Wolverhampton, Wolverhampton, WV1 1LY. I.lahart@wlv.ac.uk

Dr Anouska Carter, PhD. Senior Sports Science Manager, Faculty of Health and Wellbeing, Sheffield Hallam University, Collegiate Crescent, Sheffield, S10 2BP. A.carter@shu.ac.uk

Dr Caroline Dalton, BSc, PhD. Senior Lecturer, Faculty of Health and Wellbeing, Sheffield Hallam University, Sheffield, S10 2BP Sheffield Hallam University. C.F.Dalton@shu.ac.uk

Dr Mostafa Metwally, MD, FRCOG, MBBCh. Consultant Gynaecologist, Jessop Wing, Tree Root Walk, Sheffield S10 2SF. Mmetwally@nhs.net

Dr Markos Klonizakis, Beng (Hons), MSc, D.Phil. Reader, Faculty of Health and Wellbeing, Sheffield Hallam University, Sheffield, S10 2BP. M.klonzakis@shu.ac.uk

* Corresponding Author

The search was performed using PubMed syntax as demonstrated below, and amended for other databases:

1. “polycystic ovary syndrome”

2. PCOS

3. PCO*

4. polycystic ovar*

5. stein leventhal

6. (1 OR 2 OR 3 OR 4 OR 5)

7. Exercise[majr]

8. exercise therapy [majr]

9. Physical activity [majr]

10. Physical Fitness [majr]

11. Physical Endurance[majr]

12. exercis*[tw]

13. ((physical or motion) AND (fitness or therapy or therapies))[tw]

14. ((strength or resistance or circuit or enduran* or aerob* or physic* or fit*) AND train*)[tw]

15. (7 OR 8 OR 9 OR 10 OR 11 OR 12 OR 13 OR 14)

16. (6 AND 15)

((((((("polycystic ovary syndrome"[Title/Abstract]) OR PCOS[Title/Abstract]) OR PCO*[Title/Abstract]) OR polycystic ovar*[Title/Abstract]) OR stein leventhal[Title/Abstract]))) AND (((((((Exercise[majr]) OR exercise therapy [majr]) OR Physical activity [majr]) OR Physical Endurance[majr]) OR exercis*[tw]) OR (((physical or motion) AND (fitness or therapy or therapies))[tw])) OR (((strength or resistance or circuit or enduran* or aerob* or physic* or fit*) AND train*)[tw]))
