# Supplementary material for: The effects of physical exercise on cardiometabolic outcomes in women with polycystic ovary syndrome not taking the oral contraceptive pill: a systematic review and meta-analysis
Source: J Diabetes Metab Disord. 2019 Jul 29;18(2):597–612. doi: 10.1007/s40200-019-00425-y (PMC6915192; doi:10.1007/s40200-019-00425-y)
Supplement: Supplementary file 2 — How each judgement was reached for each category in each included study. (PDF 89 kb) [file 40200_2019_425_MOESM2_ESM.pdf]

The effects of exercise on cardiometabolic outcomes in women with polycystic ovary syndrome not taking the oral contraceptive pill: A systematic review and meta-analysis.

Journal of Diabetes and Metabolic Disorders

Ms Amie Woodward, BSc (Hons), MSc. Doctoral Researcher, Faculty of Health and Wellbeing, Sheffield Hallam University, Sheffield, S10 2BP. Amie.woodward@shu.ac.uk

Dr David Broom, PhD. Reader of Physical Activity and Health, Faculty of Health and Wellbeing, Sheffield Hallam University, Collegiate Crescent, Sheffield, S10 2BP. D.r.broom@shu.ac.uk

Deborah Harrop, BA (Hons), MA, PGCERT. Information Scientist, Nursing and Midwifery, Faculty of Health and Wellbeing, Sheffield Hallam University. D.harrop@shu.ac.uk

Dr Ian Lahart, BA (Hons), MSc, PGCERT, PhD. Senior Lecturer in Sport and Exercise Physiology. Institute of Human Science, University of Wolverhampton, Wolverhampton, WV1 1LY. I.lahart@wlv.ac.uk

Dr Anouska Carter, PhD. Senior Sports Science Manager, Faculty of Health and Wellbeing, Sheffield Hallam University, Collegiate Crescent, Sheffield, S10 2BP. A.carter@shu.ac.uk

Dr Caroline Dalton, BSc, PhD. Senior Lecturer, Faculty of Health and Wellbeing, Sheffield Hallam University, Sheffield, S10 2BP Sheffield Hallam University. C.F.Dalton@shu.ac.uk

Dr Mostafa Metwally, MD, FRCOG, MBBCh. Consultant Gynaecologist, Jessop Wing, Tree Root Walk, Sheffield S10 2SF. Mmetwally@nhs.net

Dr Markos Klonizakis, Beng (Hons), MSc, D.Phil. Reader, Faculty of Health and Wellbeing, Sheffield Hallam University, Sheffield, S10 2BP. M.klonzakis@shu.ac.uk

* Corresponding Author

Trial	Bias Domain	Source of Bias	Author's judgement	Support for judgement
Vigorito et al. 2007	Selection Bias	Random sequence generation	Unclear Risk	Not reported
		Allocation concealment	Unclear Risk	Not reported.
	Performance Bias	Blinding of participants and personnel	N/A	We did not include whether participants were blind to their allocation of intervention or to control groups, as it is often not possible (e.g. in a supervised exercise setting) to blind participants to an intervention while promoting exercise behaviour.
	Detection Bias	Blinding of outcome assessment	Low Risk	All clinical assessments were performed by the same physician who was blinded to the patient allocation.
	Attrition Bias	Incomplete outcome data	Low Risk	No attrition reported.
	Reporting Bias	Selective reporting	Unclear Risk	Unable to locate study protocol
	Other Bias	Adherence	Low Risk	All participants completed the study protocol.
Giallauria et al 2008	Selection Bias	Random sequence generation	High Risk	Allocation by patient choice
		Allocation concealment	High Risk	Patients chose allocation
	Performance Bias	Blinding of participants and personnel	N/A	We did not include whether participants were blind to their allocation of intervention or to control groups, as it is often not possible (e.g. in a supervised exercise setting) to blind participants to an intervention while promoting exercise behaviour.
	Detection Bias	Blinding of outcome assessment	Low Risk	A physician who was blinded to the patient allocation performed all clinical assessments.

	Attrition Bias	Incomplete outcome data	Low Risk	No attrition reported.
	Reporting Bias	Selective reporting	Unclear Risk	Unable to locate study protocol
	Other Bias	Adherence	Low Risk	Adherence was reported as 80%
Sprung et al 2013	Selection Bias	Random sequence generation	High Risk	Allocation by patient choice
		Allocation concealment	High Risk	Patients chose allocation
	Performance Bias	Blinding of participants and personnel	N/A	We did not include whether participants were blind to their allocation of intervention or to control groups, as it is often not possible (e.g. in a supervised exercise setting) to blind participants to an intervention while promoting exercise behaviour.
	Detection Bias	Blinding of outcome assessment	Unclear Risk	Not reported
	Attrition Bias	Incomplete outcome data	Low Risk	No attrition reported.
	Reporting Bias	Selective reporting	Unclear Risk	Unable to locate study protocol
	Other Bias	Adherence	Low Risk	91% adherence reported
